# Functional variation in a key defense gene structures herbivore communities and alters plant performance

**DOI:** 10.1371/journal.pone.0197221

**Published:** 2018-06-06

**Authors:** Nora Adam, Mario Kallenbach, Stefan Meldau, Daniel Veit, Nicole M. van Dam, Ian T. Baldwin, Meredith C. Schuman

**Affiliations:** 1 Department of Molecular Ecology, Max Planck Institute for Chemical Ecology, Jena, Germany; 2 German Centre for Integrative Biodiversity Research (iDiv), Leipzig, Germany; 3 Technical Service, Max Planck Institute for Chemical Ecology, Jena, Germany; Indian Institute of Science, INDIA

## Abstract

Plant genetic diversity structures animal communities and affects plant population productivity. However, few studies have investigated which traits are involved and the mechanisms mediating these effects. We studied the consequences of varying the expression of a single biosynthetic gene in jasmonate (JA) defense hormones, which are essential for defense against herbivores but constrain plant growth, in experimental mesocosm populations of wild tobacco (*Nicotiana attenuata*) plants under attack from three native herbivores. *Empoasca* leafhoppers preferentially attack JA-deficient *N*. *attenuata* plants in nature, and the specialist *Tupiocoris notatus* mirids avoid *Empoasca*-damaged plants. However, in experimental mesocosm populations having equal numbers of wild-type (WT) and JA-deficient plants that are silenced in the expression of the biosynthetic gene *lipoxygenase 3* (*LOX3*), *Empoasca* sp. attacked both genotypes. *Empoasca* sp. damage, rather than JA, determined *T*. *notatus* damage, which was reduced in mixed populations. The growth of specialist *Manduca sexta* larvae was reduced on WT vs. as*LOX3* monocultures, but differed in mixtures depending on caterpillar density. However, seed capsule number remained similar for WT and as*LOX3* plants in mixtures, not in monocultures, in two experimental scenarios reflecting high and low caterpillar attack. At high caterpillar density, WT plants growing in mixtures produced more seed capsules than those growing in monocultures while seed production of as*LOX3* plants did not differ by population type. However, at low caterpillar density, as*LOX3* plants growing in mixed populations produced more seed capsules than those growing in monoculture, while seed capsule production did not differ for WT by population type. Thus, mixed populations had a more stable output of seed capsules under the two scenarios. This may result from a balance between JA-mediated herbivore defense and plant competitive ability in mixed populations.

## Introduction

The diversity of traits in a species, and of the genes which control them, are the raw material for evolution by natural selection [1]. Genetic diversity in plant populations furthermore influences the composition of other trophic levels and the nature of their interactions as much as, or more than species richness, and also affects energy flow, nutrient cycling, productivity and stability of ecosystems [2–8]. However, we still have little knowledge of how intraspecific variation in plant traits might affect herbivore community dynamics and consumption by herbivores, or the consequences of increasing diversity on herbivore traits and species interactions [9]. Furthermore, most studies attempting to scale from genetic variation to effects at the community and ecosystem levels have been correlative: these have correlated or manipulated numbers of plant genotypes without quantifying the resulting trait variation; or else have manipulated traits having a complex genetic background, often without measuring underlying genetic variation [10–12]. As a consequence, we are missing links in the mechanistic chain from genetic variation, to functional traits, to community-level effects [4,13].

Recently, we argued that an approach manipulating single genes with large effects can offer greater predictive power and mechanistic understanding in trait-based studies of biodiversity, and that many important traits can be manipulated by modifying single key genes [6]. We define key genes as those which are not essential for growth and development, but regulate traits that significantly contribute to the Darwinian fitness of individuals in a particular ecological context, usually as measured by correlates like reproduction, survival, or growth. Thus key genes are precision tools for manipulating traits of ecological interest, even though the mechanisms of natural variation in a trait may differ. This approach provides explanatory power for linking intraspecific diversity to effects at other organizational levels by indicating focal measurements based on knowledge of gene function, as well as pointing to specific gene products which could be traced through interaction webs [14]. For instance, variation in a single key gene in nicotine biosynthesis in the wild coyote tobacco *Nicotiana attenuata* (Solanaceae), *putrescine methyltransferase* (*PMT*), affected herbivore, predator and pollinator communities with consequences for plant performance [15–17], and tri-trophic effects of nicotine have since been traced to specific differences in insect nicotine metabolism [18]. In this study, we use the wild tobacco *N*. *attenuata* and three of its native herbivores as an ecological model system to investigate community-level effects of variation in a key gene.

*N*. *attenuata* plants in their natural populations vary in defense traits including biosynthesis and signaling of the defense hormone jasmonate (JA) [19,20]. The generalist *Empoasca* sp. leafhopper is a piercing-sucking herbivore that feeds on phloem and cell contents. *Empoasca* sp. was observed to feed on other plant species neighboring *N*. *attenuata* plants in their native habitat. Among these plants are *Cucurbita foetidissima*, *Datura wrightii*, *Solanum americanum*, and *Mirabilis multiflora* [20,21]. Although, *N*. *attenuata* plants are an uncommon host for *Empoasca* sp. [20,21], this herbivore can feed on *N*. *attenuata* plants with reduced accumulation of the defense-related jasmonate (JA) hormones. *Empoasca* sp. females were even reported to oviposit on JA-deficient plants, as indicated by the presence of feeding, wingless nymphs [21]. *Empoasca* sp. select their host based on the plant traits perceived during the initial probing phase [20]. Therefore, *Empoasca* sp. will likely to continue feeding on plants that display the preferred traits, for example JA deficiency in *N*. *attenuata* plants or else, the highly mobile opportunistic feeder will search for other suitable host plants.

The herbivore *Tupiocoris notatus* (Distant) (Hemiptera: Miridae) is a specialist mirid bug that frequently colonizes *N*. *attenuata* plants early in the growing season as well as some other solanaceous plants, for example *Datura wrightii*, and feeds on leaf cell contents [22–26]. *T*. *notatus* females insert their eggs in the leaf midrib and into leaf veins as well as into the stem of *N*. *attenuata* plant [27]. The emerged wingless nymphs complete their life cycle on *N*. *attenuata* plants and both adults and nymphs are highly mobile within the plant [28]. This specialist preferentially feeds on younger leaves of the plant [29]. Life history traits of *T*. *notatus* are not directly affected by host plant JA deficiency [27,30]. However, *T*. *notatus* damage is reduced on *N*. *attenuata* plants infested by the generalist leafhopper *Empoasca* sp. [30]. Although *T*. *notatus* is one of the most abundant herbivores on *N*. *attenuata* plants, its damage does not significantly affect plant fitness in nature [26]. In contrast, the chewing larvae of the Solanaceous specialist *Manduca sexta* (Lepidoptera, Sphingidae) are among the most damaging insect herbivores attacking *N*. *attenuata* plants, and these larvae grow larger on JA-deficient plants [31]. *Manduca sexta* moths avoid specific plant volatiles which are regulated by jasmonic acid [25]. This would indicate that caterpillar density on as*LOX3* plants might be high [25]. Also, *Manduca sexta* larvae may have increased mortality and decreased oviposition on plants infested by *T*. *notatus* in nature [26].

We hypothesized that varying the production of JA within experimental *N*. *attenuata* populations by manipulating a single key biosynthetic gene would alter feeding damage and performance of these three native herbivores and consequently plant fitness outcomes. We tested this hypothesis in two mesocosm experiments, where we exposed monocultures of either wild-type (WT) plants or of plants rendered deficient in the JA biosynthetic gene *Lipoxygenase 3* by RNAi (antisense, as*LOX3*), and a mixed culture comprising equal numbers of WT and as*LOX3*, to different herbivory regimes. We then measured herbivore damage and performance, and plant growth and reproduction. We choose *LOX3* as a key gene in the JA pathway, since plants abrogated in its expression produce ca. 50% the JA concentration of WT plants, reflecting natural diversity in JA production [19,20]. Furthermore, this gene showed large effects on plant productivity and herbivore performance, and multiple as*LOX3* lines have been characterized and shown to vary only in their JA production and resulting effects [21,31–33].

We predicted that mixtures would receive damage from *Empoasca* sp. intermediate between WT monocultures (low damage) and as*LOX3* monocultures (high damage). We furthermore predicted that *Empoasca* sp. damage would be a better predictor of *T*. *notatus* damage than plant genotype [27,30], with the alternative hypothesis being that plant genotype was in fact a better predictor. For *M*. *sexta* larval growth, we similarly predicted that either plant genotype would be a better predictor of *M*. *sexta* growth than damage from the other two herbivores [31], or that *T*. *notatus* damage would be a better predictor than plant genotype if *T*. *notatus* had strong effects on *M*. *sexta* performance in the absence of predators [26]. Finally, we also had two non-exclusive hypotheses for plant fitness outcomes: that they would be determined primarily by *M*. *sexta* attack patterns; and that fitness correlates would be more consistent for mixtures than monocultures as a result of the balance between the expected defense advantage of WT plants, and the known growth advantage of as*LOX3* plants [31,32]. From our data, we conclude that variation in a single key defense gene could have community-level effects by altering damage patterns by native herbivores, caterpillar performance, and, as a consequence, determining plant fitness outcomes.

## Materials and methods

### Mesocosm experimental design

**Fig 1:**
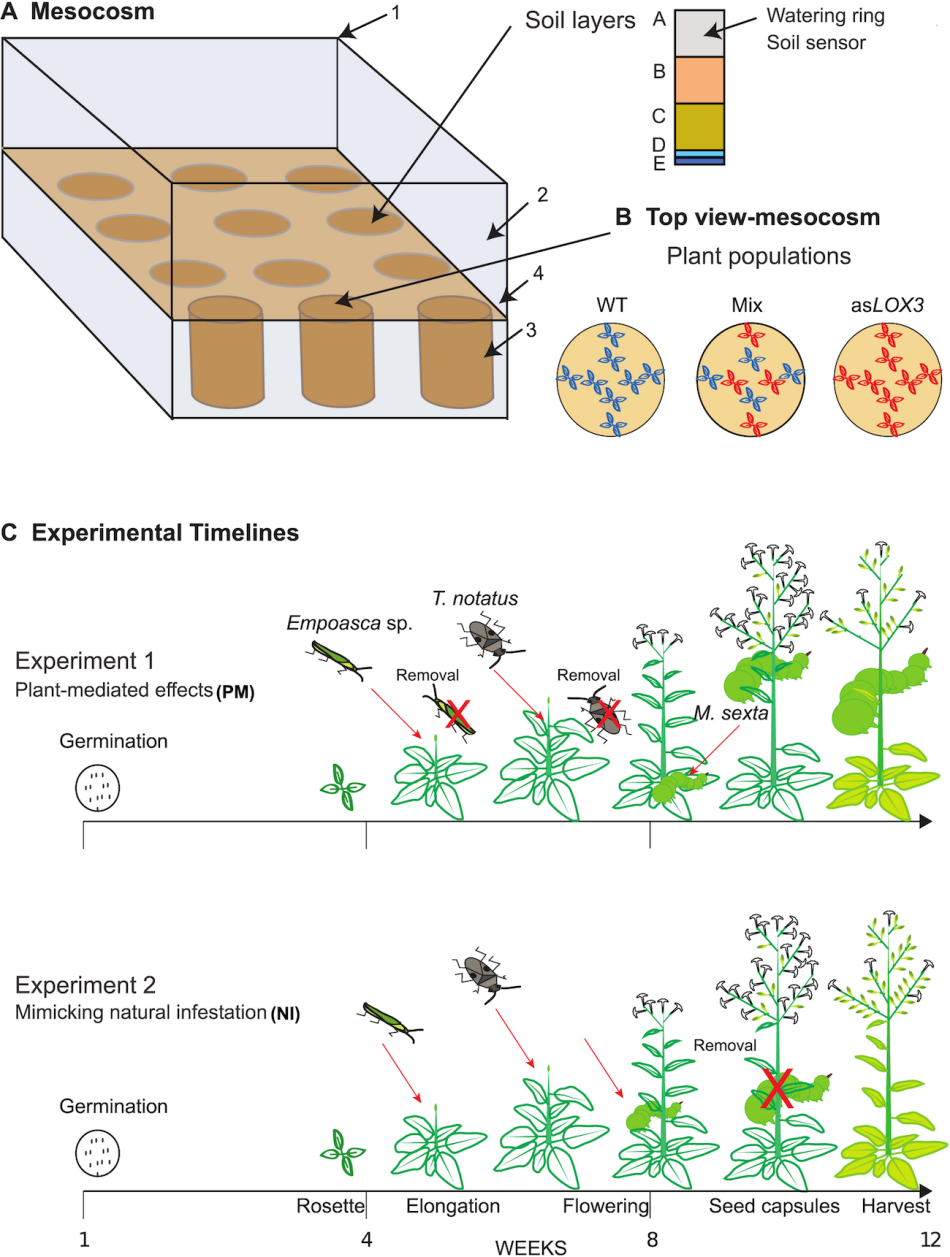
Experimental design. (A) The experimental mesocosm (4 x 3 x 2.5 m l x w x h) consists of 12 containers within a screen- and plastic-enclosed box in a glasshouse. Aluminum containers filled with soil (1, RSonline), a stainless steel 0.8 mm mesh frame (2, Scherer Insektenschutz), and a polypropylene container and plates (3 and 4, HL Kunststofftechnik) were used to build the mesocosm. *Nicotiana* substrate and other substrates are described in **Tables A and B in S1 File**. (B) Eight *N*. *attenuata* plants from either the same genotype (monoculture) or containing four of each genotype (mixed culture) were planted in each mesocosm container. (C) The experimental timeline shows the differences between Experiment 1 (Plant-mediated, PM) and Experiment 2 (Natural infestation, NI), including all other experimental steps (germination, transfer to the glasshouse, planting, herbivore infestation and data collection of growth and fitness parameters). Xiang Li, flower and seed capsule icons.

To test the consequences of varying JA deficiency in plant populations under attack by three native herbivores, two experiments were performed in a glasshouse mesocosm. The glasshouse mesocosm reflects a semi-natural condition, where herbivores can choose to move among different plant populations. The two experiments were designed to reflect two possible ecological scenarios, where WT and JA-deficient as*LOX3* plants were grown as replicated monocultures, and mixtures comprising equal numbers of each genotype (Fig 1A, Fig 1B). The mesocosm has a total of 12 containers allowing for a total of four replicates per culture type (mixture, WT and as*LOX3* monocultures) in each experiment. In the two experiments, initially eight plants from either the same genotype (monocultures) or comprising four of each genotype in an alternating pattern (mixture; Fig 1B), were planted together per one mesocosm container, forming the three culture types. We initially planted a total of 96 plants in each mesocosm experiment, where *n* = 8 plants were planted in one mesocosm container. Of 96 plants initially planted in each experiment, 15 and 7 individual plants distributed in different cultures were excluded from the analyses in the PM- and NI Experiments, respectively, because these plants were not healthy (stunted growth), did not survive to elongation or flowering stage, or had cumulative counts which decreased over time, which was attributed to measurement errors, and therefore, we had *n* = 4–8 plants/culture type for the statistical analysis. When either one WT or as*LOX3* plant had to be excluded from the mixtures, a randomly chosen plant from the other genotype was also excluded to balance genotype frequency. This ensured that results of the mixtures are not a product of unequal numbers of the genotypes.

In the two mesocosm experiments, three native herbivores were added according to the order of their natural appearance observed over more than 10 years of field work in the portion of their native habitat in southwestern Utah: adult *Empoasca* sp. were allowed to disperse in the mesocosm once the majority of plants were elongating (n = 5/plant, but released not onto plants directly but at three points evenly distributed across the mesocosm); then adults of *T*. *notatus* were added one week later (n = 5/plant, but again not directly to plants but released at three points distributed across the mesocosm); and finally, freshly hatched neonates of *M*. *sexta* were added to mimic oviposition (n = 2/plant directly to 4 plants/population, i.e., every second plant in a population), once the majority of plants flowered. Both *Empoasca* sp. and *T*. *notatus* were released in three standard positions distributed evenly among the 12 mesocosm containers and not directly over any container, in the center and at the edges of the mesocosm. This allows an equal initial distribution of herbivores on the different plant populations.

#### Plant-mediated effects

In Experiment 1, we investigated **Plant-Mediated (PM)** effects by removing the first two herbivores after each had fed alone on the plant populations. Herbivore species might interact directly [34], and the removal of each species before adding the subsequent species avoided direct interactions between herbivore species. Here, infestations with *Empoasca* sp. proceeded for ca. one week and then insects were collected and removed from the experiment. *T*. *notatus* adults were then added until a majority of plants flowered, at which time they also were collected and removed after ca. one week of infestation. Damage from each herbivore was estimated upon removal. It should be noted that we cannot completely exclude that a few individuals were overlooked during removal. We also cannot exclude that the insects, specifically the specialist *T*. *notatus*, laid eggs on plants. Thus to avoid unwanted herbivore presence, we regularly monitored for nymphs. Our removal strongly minimizes effects from direct herbivore interaction by drastically reducing the population of each species in the experimental design. Larvae of *M*. *sexta* were added to the plants after simulated oviposition (distribution of neonates) and because it is generally the last herbivore species to arrive and was the last species added, larvae of *M*. *sexta* were allowed to remain until they consumed large portions of plants in the mesocosm (the majority of caterpillars were close to pupation at this stage). Caterpillars were allowed to move between plant populations of their own volition, reflecting *M*. *sexta* outbreaks and allowing simulation of a high rate of attack *M*. *sexta* larvae, the most damaging of these herbivores for the plant. At this high attack rate, *M*. *sexta* larvae began to move between populations after approximately two weeks.

#### Natural infestation

In Experiment 2 **(Natural Infestation, NI**), we mimicked the more common natural situation of herbivore co-occurrence, allowing all herbivores to remain throughout the experiment, permitting co-occurrence. Since we simulated unusually (but not unprecedented) high rates of attack by *M*. *sexta* larvae in the PM Experiment, we were also interested in simulating a more common, lower rate of attack in the NI Experiment. For this reason, *M*. *sexta* larval density was reduced after 9 days of feeding to *n* = 3/population, once larvae became mobile between plants to mimic natural dispersal outside of the small area defined by the mesocosm or population reduction due to predators, and all caterpillars were removed after 12 days [35]. This reduction restricted the movement of remaining larvae so that they remained within plant populations and did not wander between populations.

### Plant material and growth conditions

Seeds of wild-type (WT, 31^st^ inbred generation) *Nicotiana attenuata* Torr. Ex.Watts. (Solanaceae) and the third transformed generation (T3) of a transgenic line with reduced levels of JA (as*LOX3*, homozygous harboring a single T-DNA insertion, line number A-300 [31]) were used in this study. The WT line was generated from seeds collected in 1988 from a natural population at the DI Ranch in southwestern Utah [23]. In both experiments, germination was performed as previously described [36,37] except that in the NI Experiment, seeds were first incubated for 3 d at 30°C and then 7 d at 26°C to counteract seasonal fluctuations in dormancy. After 10 d, seedlings were transferred to Teku pots and kept under glasshouse conditions as previously described [36,37]. Twelve to thirteen days later, plants were transferred to an experimental mesocosm (Fig 1) in the remote Isserstedt glasshouse of the Max Planck Institute for Chemical Ecology in Jena, Germany, used for experiments with highly mobile insects; and planted into 12 mesocosm containers (Fig 1A; Polypropylen, HL Kunststofftechnik, Ø700 x 1000 mm). The plants were planted into the top of four soil layers (**Tables A, B, C in S1 File**). Soil moisture and temperature in the mesocosm were also monitored (see **S2 File**). Day- and nighttime temperatures in the glasshouse were 26–28°C and 20–22°C, respectively, as previously described [36]. The photoperiod was a constant 16 h (6:00–22:00) consisting of natural photoperiods (latitude 50°N) with day extension lighting provided by high-pressure sodium (HPS) lamps delivering a photosynthetic photon flux (PPF) of 160 μmol m^-2^ s^-1^. Plants were watered with 1.2 L/population every second day using an automated drip irrigation system. The irrigation system consisted of 12 pumps (Wilhelm Keller GmbH & Co. KG), one for each container, controlled with a Siemens LOGO (RSonline). Each pump was connected with a tube (PUN 6 x 4 mm) to a check valve (Jenpneumatik), which was connected to a dripping ring (Conrad). A polypropylene cross (HL-Kunststofftechnik) was placed over the ring to stabilize its form. When plants elongated, watering was increased to 2 L/population on the same schedule. Plants were fertilized once a week with Peters Allrounder (1 g/L water, **Table C in S1 File**). Soil moisture and temperature were monitored throughout experiments (**S2 File**).

In a preliminary experiment conducted in small cages, prior to the mesocosm experiments, plants were germinated and grown as described for the PM Experiment except that single plants were planted in 4 L pots and transported to the remote Isserstedt glasshouse 10 d after transfer to Teku pots, and were watered and fertilized by hand.

### Insects

*Empoasca* sp. (Hemiptera: Cicadellidae) and *Tupiocoris notatus* Distant (Hemiptera: Miridae) were collected from Lytle Preserve in southwestern Utah. *Empoasca* sp. was collected from *Cucurbita foetidissima* and *T*. *notatus* from *N*. *attenuata* plants. Both species were maintained in a colony in the Isserstedt glasshouse as previously described [20,26]. *Manduca sexta* Linnaeus (Lepidoptera: Sphingidae) eggs were collected from an in-house colony at the Max Planck Institute for Chemical Ecology in Jena. Collected eggs were incubated in a growth chamber (Snijders Scientific) at 26°C/16 h light, 24°C/8 h darkness and 65% relative humidity. Freshly hatched neonates were used for caterpillar growth assays.

### Pre-mesocosm experiment: *T*. *notaus* and *Empoasca* sp. interaction

To analyze the interaction between *T*. *notaus* and *Empoasca* sp. in small scale and more controlled conditions, we conducted an experiment prior to the mesocosm experiments in which one genotype (WT or as*LOX3*) was exposed to either *T*. *notatus* alone, or *T*. *notatus* and *Empoasca* sp. under glasshouse conditions. In total, seven plants of each genotype (WT or as*LOX3*) were used in this experiment. The seven plants formed a total of four replicates of each genotype per herbivore treatment, where either two plants or one lone plant of the same genotype were caged together. Plants then were exposed to herbivore treatment by either simultaneous exposure to adults of *T*. *notatus* and *Empoasca* sp., or only to *T*. *notatus* (10 insects/herbivore species/plant), for one week. The infestation was repeated for another two weeks. In the last week, only 5 insects/species/plant were added to each cage, for a total of 25 insects/species/plant added over the course of the experiment. To determine *T*. *notatus* and *Empoasca* sp. damage, high resolution pictures of 15 leaves per plant in standardized rosette, stem, and young leaf positions were taken. Damage was evaluated with Photoshop and expressed as a percentage of total damage caused to the plant. *Empoasca* sp. and *T*. *notaus* have distinct damage characteristics that make it possible to distinguish damage caused by each herbivore [20,30]. For example, *Empoasca* sp. damage is characterized by appearing of small pale green to white dots that are often connected on the upper side of the leaf. *T*. *notatus* damage appears as relatively faded regions on the upper side of the leaf with distinct black dots on the underside of the leaf (**Fig A in S1 File**).

### Herbivory damage and caterpillar growth assay

Each plant was carefully checked for feeding damage based on the damage characteristic of *Empoasca* sp. and *T*. *notatus*, and leaf area removal by *M*. *sexta* caterpillars. Damage was estimated on a scale with single percentage point gradations, except for a few plants with very low damage, where finer gradations below one percentage point were used. The percentage values were derived from visual damage assessment by counting damaged leaves and estimating their area fraction of the total plant similarly to previous studies [6,38]. All damage estimates were performed by N. A. Damage caused by *Empoasca* sp. and *T*. *notatus* was estimated after ca. one week of infestation in both experiments. Freshly hatched *M*. *sexta* neonates were added in standardized positions to one rosette and one lower stem leaf at 63 and 54 d post-germination in the PM- and NI Experiments, respectively. Differences in absolute number of days of herbivore treatment between experiments correspond to differences in plant growth. Physiological processes such as elongation and flowering were used as indicators to determine infestation time, rather than absolute number of days post-germination. Caterpillar mass was measured at days 5, 8, 11 and 15 post-addition in the PM Experiment, and at days 5, 9 and 12 post-addition in the NI Experiment, using a laboratory balance (METTLER AE100, ±0.1mg). Damage caused by *M*. *sexta* larvae was estimated only in the NI Experiment, on days 10 and 12 post-infestation, to have a final measure of their damage. Cumulative damage data from day 12 were then used for statistical analysis.

### Plant growth and fitness parameters

Maximum rosette diameter was measured in the PM Experiment on days 30, 34 and 38 post-germination until elongation, by laying a ruler over the rosette. In the NI Experiment, we measured rosette diameter once plants had elongated (day 41). Plant height was measured on days 34, 38 and 52 (PM Experiment) and days 41, 53,63 (NI Experiment) post-germination, respectively, from the stem base to the tip of the apical inflorescence by placing a measuring stick near the stem. Once plants began flowering, the number of open flowers per plant was counted on days 44, 52, 60, 70 and 75 post-germination in the PM Experiment, and days 46, 56, 66, 74 and 81 in the NI Experiment. Numbers of ripe seed capsules per plant were counted at three time points in the PM Experiment (days 63, 70 and 75). In the NI Experiment, the number of ripe seed capsules per plant was counted until all plants had senesced and no new flowers were produced (on days 60, 66, 74 and 81). Plants were harvested when large portions of whole plant populations were consumed by *M*. *sexta* larvae (PM Experiment), or when most plants had senesced (NI Experiment). Plant fresh weight and dry weight was measured at harvest.

### Statistical analyses

Data were evaluated at population and genotype levels. Population level refers to data collected from all monitored plants in one mesocosm container. The genotype level represents data collected from either WT or as*LOX3* plants growing in mixed culture and compared to data collected from the corresponding genotypes growing in monoculture. All statistical tests were performed with R (R-Project; http://www.r-project.org, [39]). To account for pseudo-replication in the design, data were analyzed either with linear mixed effects models [40] or by using the mean value of observations from one mesocosm container (population). ANOVAs followed by Tukey HSD *post-hoc* tests were used when model assumptions were met. Non-parametric data were analyzed with Kruskal-Wallis rank sum tests followed by Dunn's test for multiple comparisons, or generalized least squares models (GLS). All damage data were arcsine transformed.

## Results

### *Empoasca* sp. caused similarly high damage to as*LOX3* monocultures and mixtures, determining *T*. *notatus* damage patterns

**Fig 2:**
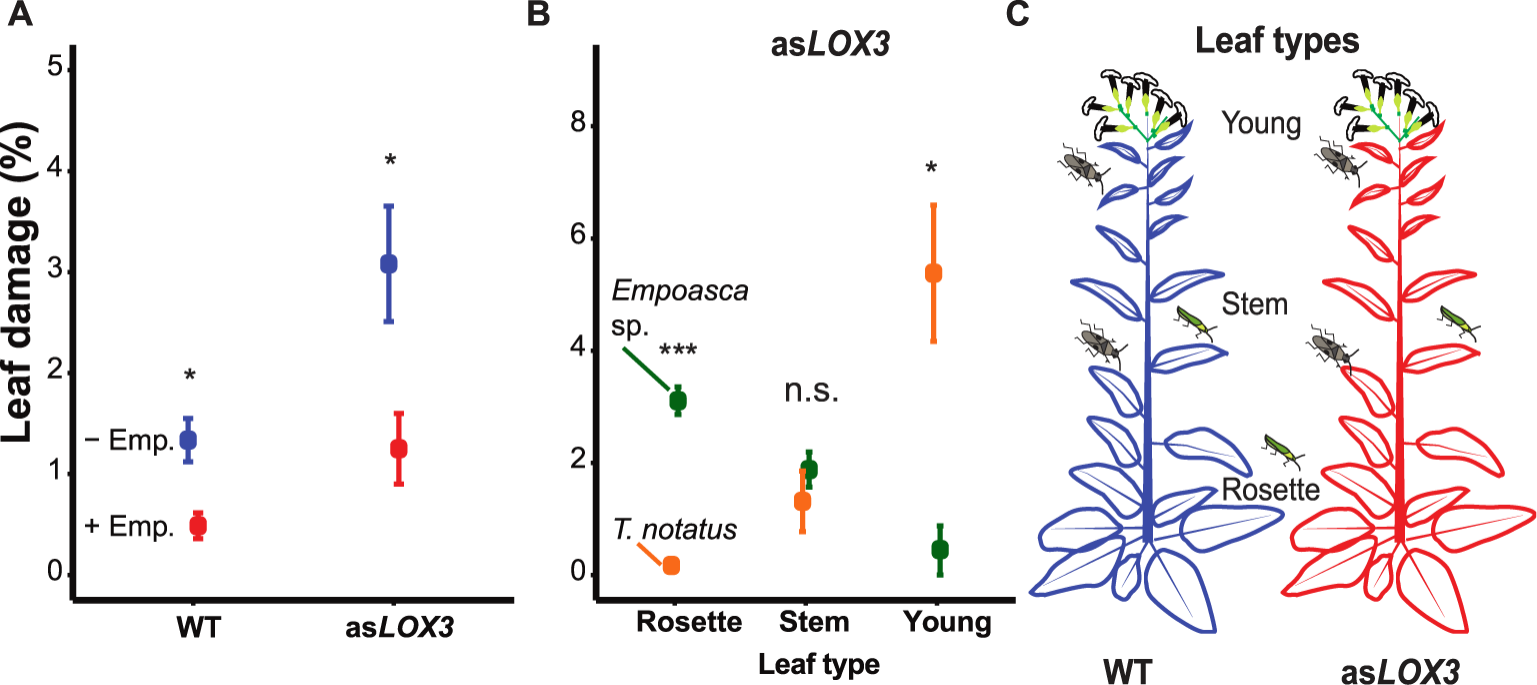
*T*. *notatus* causes less damage on *Empoasca* sp. co-infested *N*. *attenuata* plants (WT and as*LOX3*) and these herbivores have opposite preferences for young and old leaves. Values are mean±SE. (A) *T*. *notatus* caused significantly less damage on *Empoasca* sp. co-infested plants on WT and as*LOX3* plants. (B) *Empoasca* sp. feeds generally more on as*LOX3* and caused more damage to as*LOX3* rosette leaves than *T*. *notatus*; the opposite pattern was observed on young leaves (data are for herbivores feeding on as*LOX3 N*. *attenuata* plants). (C) Description of within-plant damage distribution and leaf positions on as*LOX3 N*. *attenuata* plants attacked by the herbivores. Damage data were arcsin square root transformed. *P < 0.05 in Student’s t-tests between treatments (+/- *Empoasca* sp.) within each genotype, ***P < 0.001 in Student’s t-tests and *P < 0.05 in Wilcox test between insects within each leaf type. n.s. = not significant.

In a preliminary experiment (see pre-mesocosm experiment: *T*. *notaus* and *Empoasca* sp. interaction), *T*. *notatus* damage was reduced when plants were co-infested with *Empoasca* sp. (Student’s t-tests within each genotype, df = 6, t = 3.569, P = 0.011, *d* = 2.523 and t = 2.661, P = 0.037, *d* = 1.881 for WT and as*LOX3*, respectively; Fig 2A). Furthermore, *Empoasca* sp. and *T*. *notatus* showed contrasting preferences for young and old leaves on as*LOX3* plants (Student’s t-tests and Wilcox test between insects within each leaf type; df = 6, t = -8.996, P < 0.001, *d* = 6.361, t = -1.113, P = 0.308, *d* = 0.787, W = 16, P = 0.026, *d* = 3.181 for rosette, stem and young leaves, respectively; Fig 2B, Fig 2C).

**Fig 3:**
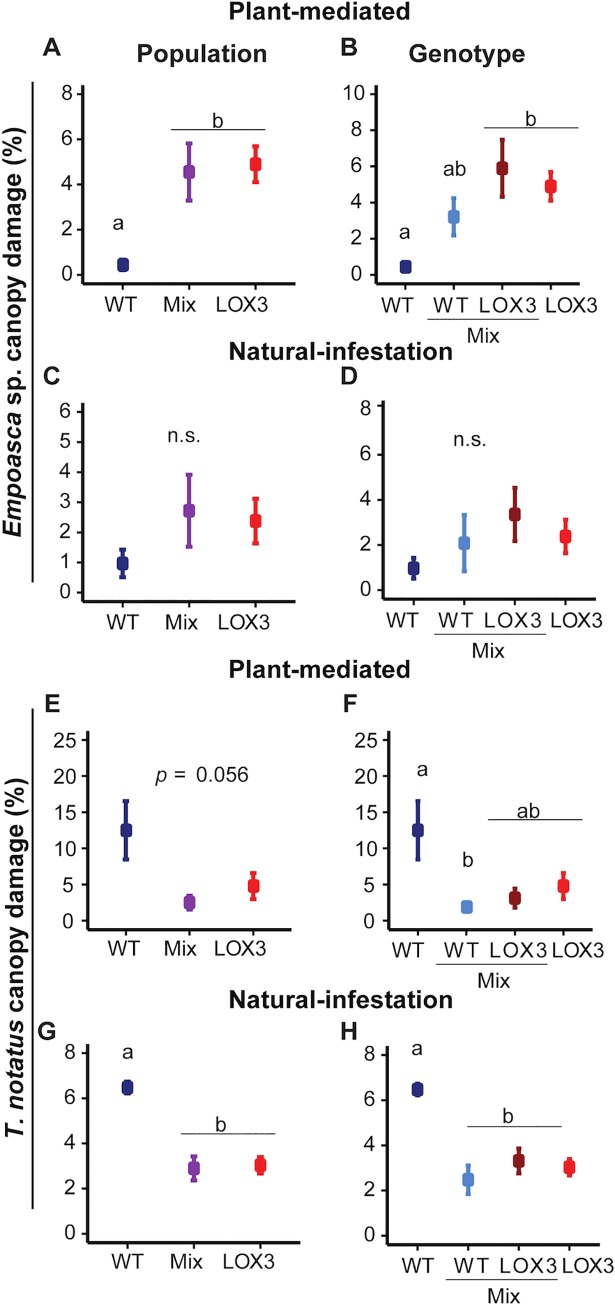
*Empoasca* sp. caused more damage to as*LOX3* mono- and mixtures compared to WT monocultures, determining *T*. *notatus* damage patterns. Values are mean±SE. (A) In the PM Experiment, plants in WT monocultures received less *Empoasca* sp. damage than plants in mixtures and as*LOX3* monocultures. (B) At the genotype level, as*LOX3* plants growing either in the mixed or in the monoculture were damaged at a similar rate by *Empoasca* sp. but WT plants in the mixed culture tended to be more attacked by *Empoasca* sp. than those growing in the monoculture. (C, D) In the NI Experiment, *Empoasca* sp. damage showed similar patterns as in the PM Experiment but the damage rate did not differ significantly among populations and genotypes. (E, G) In both experiments, *T*. *notatus* caused more damage to plants in WT monocultures in comparison to plants in mixtures and as*LOX3* monoculture. (E, F) In addition, *T*. *notatus* caused significantly more damage to WT plants growing in monocultures than to WT plants growing in mixtures. The amount of damaged received by as*LOX3* plants did not differ when plants were growing in mixed or in monocultures. Different letters indicate statistically significant differences in Tukey HSD tests following a significant one-way ANOVA (P < 0.01) or rank sum Dunn’s tests following a significant Kruskal-Wallis test (P < 0.05); n.s. = not significant; n = 4; one-way ANOVA (*P* = 0.056, A).

In the mesocosm, and at the population level, as*LOX3* monocultures and mixtures received significantly more damage from *Empoasca* sp. than did WT monocultures in the PM Experiment (Table 1, Fig 3A). At the genotype level, WT plants in monocultures received less damage than as*LOX3* plants and WT plants in mixtures, but this difference was not statistically significant for the NI experiment (Table 1, Fig 3B).

**Table 1:** Statistical summary for *Empoasca* sp. damage.

Herbivore	Experiment	Level	Test	Test-statistic	*df*	Pairwise test	Comparison	*P*	*d*
*Empoasca* sp.	PM	Population	ANOVA	*F* = 13.56	2,9	TukeyHSD	asLOX3 vs Mix	0.577	0.679
							asLOX3 vs WT	**0.002**	**4.167**
							Mix vs WT	**0.009**	**2.579**
		Genotype	ANOVA	*F* = 9.684	3,12	TukeyHSD	asLOX3_mono vs asLOX3_mix	0.999	0.079
							asLOX3_mono vs WT_mono	**0.003**	**4.167**
							asLOX3_mix vs WT_mono	**0.002**	**3.048**
							asLOX3_mono vs WT_mix	0.231	1.588
							WT_mix vs WT_mono	0.103	1.995
							WT_mix vs asLOX3_mix	0.192	1.285
	NI	Population	ANOVA P > 0.3	*F* = 1.151	2,9				
		Genotype	ANOVA P > 0.4	*F* = 0.932	3,12				

In contrast, *Empoasca* sp. attacked as*LOX3* plants at similar rates regardless of whether they were growing in monoculture or mixture, whereas damage on the two plant genotypes in mixtures was similar (Table 2, Fig 3B). Results from the NI Experiment for *Empoasca* sp. damage at the genotype level showed similar trends as in the PM Experiment (Table 2, Fig 3C, Fig 3D) but the damage rate did not differ significantly and was generally lower than in the PM Experiment.

**Table 2:** Statistical summary for *T*. *notatus* sp. damage.

Herbivore	Experiment	Level	Test	Test-statistic	*df*	Pairwise test	Comparison	*P*	*d*
*T*. *notatus*	PM	Population	ANOVA P = 0.056	*F* = 4.038	2,9				
		Genotype	ANOVA	*F* = 4.048	3,12	TukeyHSD	asLOX3_mono vs asLOX3_mix	0.991	0.194
							asLOX3_mono vs WT_mono	0.162	1.288
							asLOX3_mix vs WT_mono	0.102	1.838
							asLOX3_mono vs WT_mix	0.725	0.727
							WT_mix vs WT_mono	**0.027**	**2.481**
							WT_mix vs asLOX3_mix	0.871	0.789
	NI	Population	Kruskal-Wallis	*X*^*2*^ = 7.423	2	Dunn's-test	asLOX3 vs Mix	0.845	0.279
							asLOX3 vs WT	**0.024**	**4.759**
							Mix vs WT	**0.014**	**3.588**
		Genotype	ANOVA	*F* = 9.676	3,12	TukeyHSD	asLOX3_mono vs asLOX3_mix	0.992	0.194
							asLOX3_mono vs WT_mono	**0.008**	**4.759**
							asLOX3_mix vs WT_mono	**0.013**	**3.024**
							asLOX3_mono vs WT_mix	0.716	0.673
							WT_mix vs WT_mono	**0.001**	**3.586**
							WT_mix vs asLOX3_mix	0.556	0.738

In contrast to *Empoasca* sp., *T*. *notatus* caused more damage to WT monocultures than to as*LOX3* mono- and mixed cultures, which had similarly low damage rates (Table 2, Fig 3E, Fig 3G).

At the genotype level, *T*. *notatus* caused more damage on WT growing in monocultures compared to WT in mixtures, in both experiments. Damage caused to WT growing in monocultures was higher than damage on as*LOX3* plants regardless of the culture identity in the NI Experiment, and did not differ statistically in the PM Experiment. Damage caused to both genotypes in mixture was similar to each other and to damage on as*LOX3* plants growing in the monocultures (Table 2, Fig 3F, Fig 3H).

### *M*. *sexta* caterpillars grew smaller and tended to cause less damage on WT monocultures compared to as*LOX3* monocultures, while mixtures showed mixed results

**Fig 4:**
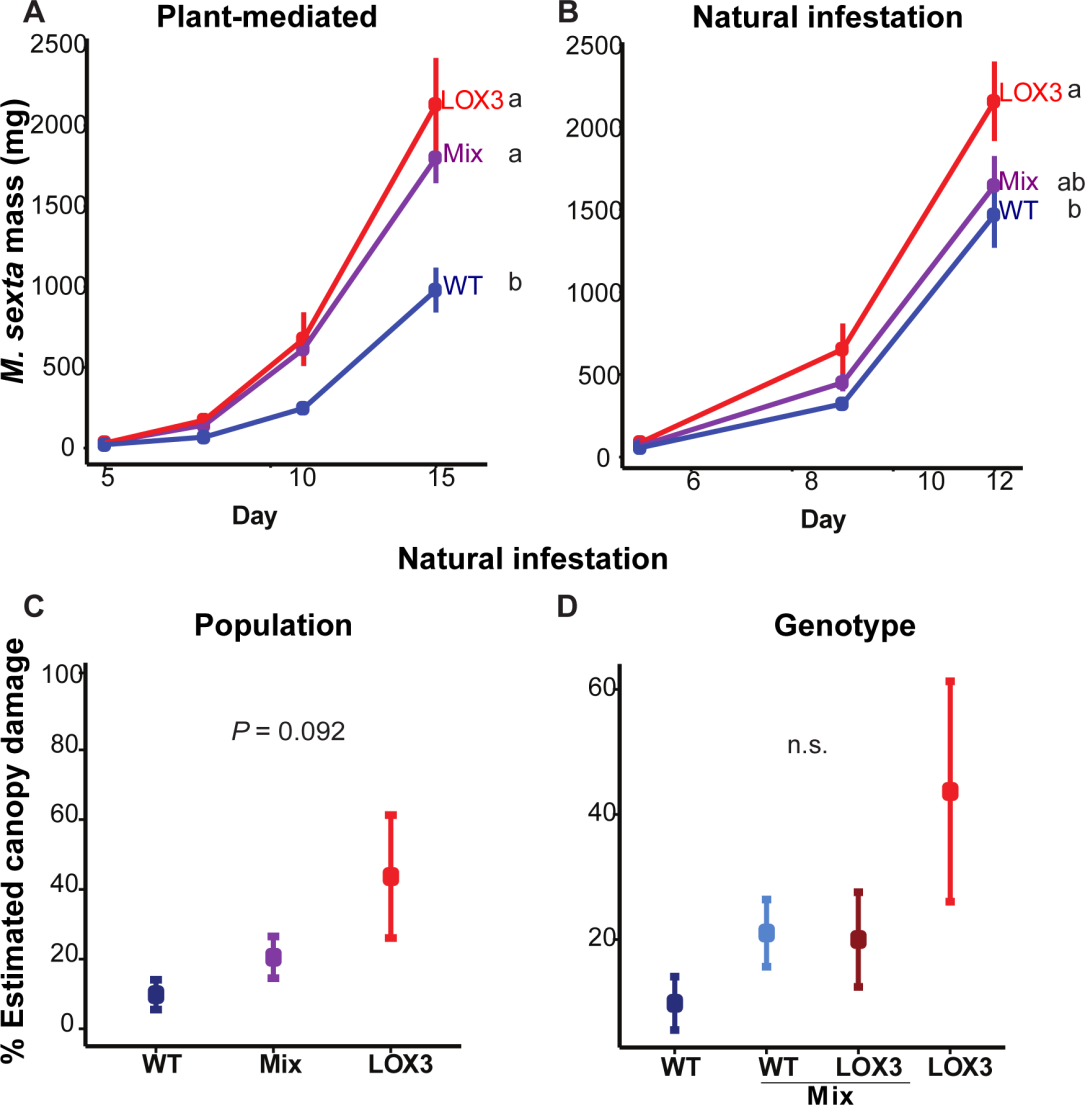
*M*. *sexta* caterpillars grew smaller on WT monocultures and tended to consume less leaf area than on as*LOX3* monocultures. Values are mean±SE. (A) In the PM Experiment, caterpillars were larger on plants in mixtures and as*LOX3* monocultures than on WT monocultures. (B) In the NI Experiment, caterpillar growth was consistent with results of the PM Experiment for WT and as*LOX3*. However, the growth on mixtures was intermediate. (C) Estimated consumed leaf area by caterpillar feeding on WT monocultures tended to be lower than on as*LOX3* mono- and mixtures. (D) The estimated consumed leaf area did not differ between as*LOX3* or WT plants growing in mixed culture when compared to the corresponding genotypes in monocultures. Different letters indicate statistically significant differences; n.s = not significant; linear-mixed-effects-model; GLS; one-way ANOVA (*P* = 0.092, C).

In both experiments, caterpillars grew smaller on WT monocultures in comparison to as*LOX3* monocultures (linear mixed-effects model, Table 3, Fig 4A, Fig 4B). However, larval growth showed different results in mixtures between experiments. While growth in mixtures differed from WT, but not from as*LOX3* monocultures in the PM Experiment, it was intermediate in the NI Experiment (Table 3, linear mixed-effects model, Fig 4A; GLS, Fig 4B).

**Table 3:** Statistical summary for *M*. *sexta* growth.

Parameter	Experiment	Level	Test/Model	Comparison	n	*P*	*d*
Weight	PM	Population	LME	asLOX3 vs Mix	4	0.305	0.176
				asLOX3 vs WT	4	**0.0007**	**2.517**
				Mix vs WT	4	**0.011**	**2.724**
		Genotype	LME	asLOX3_mono vs asLOX3_mix	4	0.249	0.732
				asLOX3_mono vs WT_mono	4	**0.016**	**1.608**
				asLOX3_mix_ vs WT_mono	4	**0.001**	**4.482**
				asLOX3_mono vs WT_mix	4	**0.010**	**1.666**
				WT_mix vs WT_mono	4	0.836	0.190
				WT_mix vs asLOX3_mix	4	**0.0009**	4.148
	NI	Population	GLS	asLOX3 vs Mix	4	0.231	1.517
				asLOX3 vs WT	4	**0.023**	**1.485**
				Mix vs WT	4	0.225	0.438

The growth of caterpillars depended on the identity of the plant genotype rather than on population identity. For example, the growth of caterpillars feeding on as*LOX3* plants in mixed versus monocultures did not differ (linear mixed-effects model, Table 3, **Fig B in S1 File**).

WT monocultures tended to receive less damage by *M*. *sexta* caterpillars than mixed and as*LOX3* monocultures, but the differences were not statistically significant (Kruskal-Wallis rank sum test, *X*^2^ = 4.769, P = 0.092; Fig 4C). Feeding damage from *M*. *sexta* did not differ significantly among genotypes (Kruskal-Wallis rank sum test, *X*^2^ = 5.417, P = 0.143; Fig 4D).

### Mixtures were more stable in terms of seed capsule production across experiments

Final rosette diameter and height did not differ by population or genotype in either experiment, nor did fresh or dry biomass (**Table D in S1 File**). In both experiments, flower and seed capsule number was counted throughout the reproductive period as a measure of realized reproduction and an indicator of plant Darwinian fitness.

**Fig 5:**
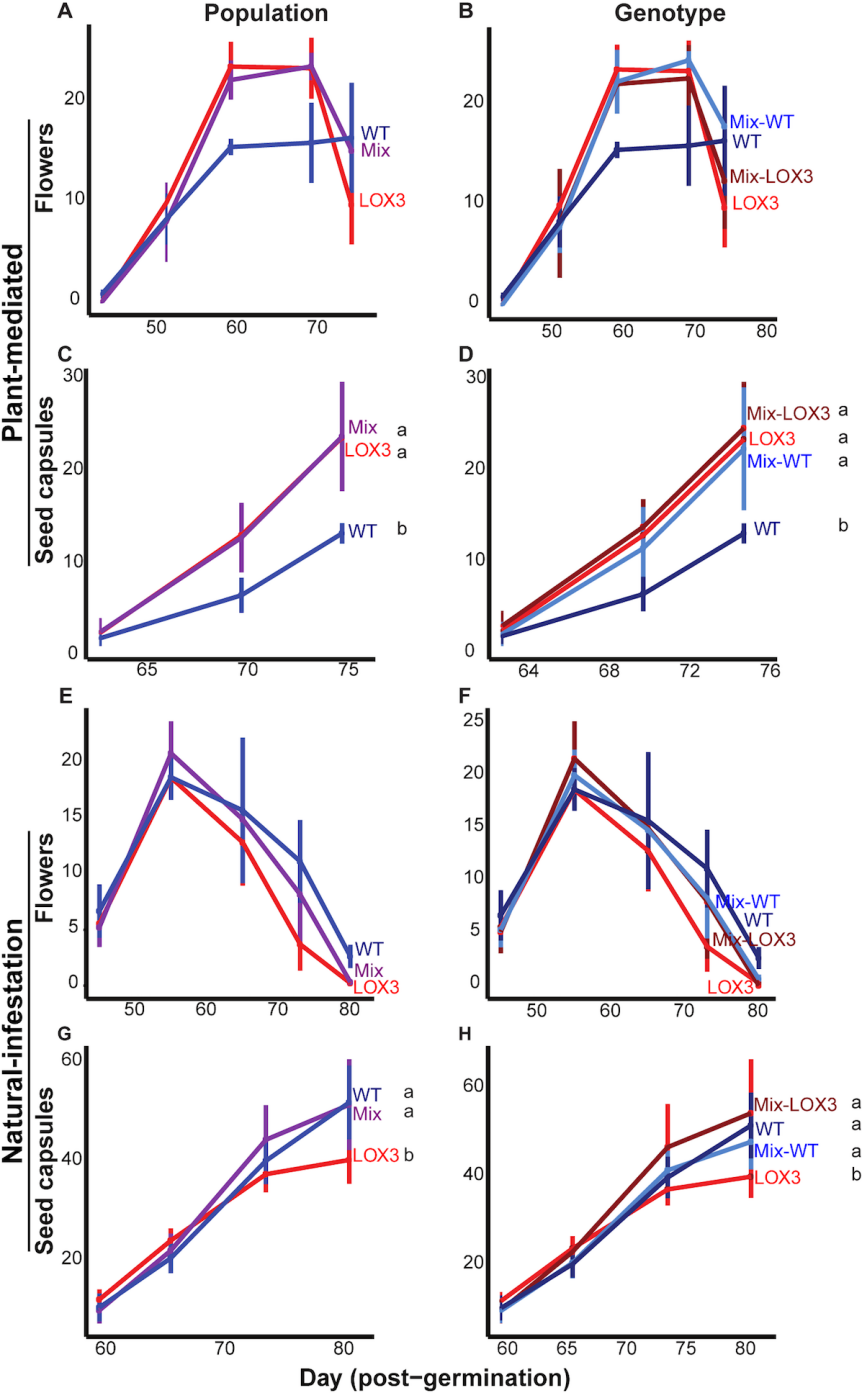
Mixtures were more stable in terms of seed capsule production across experiments and number of flowers did not differ between populations. Values are mean±SE. (A) At higher caterpillar densities (PM Experiment), WT monocultures tended to produce fewer flowers than other population types. (B) WT growing in the mixed culture tended to have more flowers than those growing in monocultures, while the number of flowers produced by as*LOX3* plants growing either in mono- or mixtures did not differ. (C) WT monocultures produced fewer seed capsules than as*LOX3* mono- and mixtures when caterpillar density was high. (D) At the genotype level, WT plants growing in monocultures produced fewer seed capsules than those growing in mixtures, while the number of seed capsules did not differ between as*LOX3* plants when growing in mono- or in mixtures. (E, F) When the caterpillar density was reduced and later all caterpillars were removed from the mesocosm (NI Experiment), the number of flowers did not differ between populations or genotypes. (G) as*LOX3* monocultures produced fewer seed capsules than mixed and WT monocultures. (H) At the genotype level, as*LOX3* plants growing in the monoculture had fewer seed capsules than those growing in the mixed culture while the number of seed capsules did not differ between WT plants growing in monoculture or mixed culture. Different letters indicate significant differences; n = 4; linear mixed-effects models; see Table 4 for complete analysis.

In both experiments, the number of flowers did not differ significantly by population or by genotype, but there was a tendency for WT plants in monocultures (population and genotypic level) to produce fewer flowers than as*LOX3* monocultures and mixtures in the PM Experiment (linear mixed-effects model, Table 4, Fig 5A, Fig 5B, Fig 5E, Fig 5F).

**Table 4:** Statistical summary for flowers and seed capsules.

Experiment	Parameter	Level	Comparison	*n*	*P*	*d*
PM	Flowers	population	Mix vs asLOX3	4	0.175	
			WT vs asLOX3	4	0.425	
			WT vs Mix	4	0.547	
		genotype	asLOX3-mono vs asLOX3-mix	4	0.507	
			asLOX3-mono vs WT-mix	4	0.129	
			WT-mono vs asLOX3-mix	4	0.958	
			WT-mono vs WT-mix	4	0.361	
			asLOX3-mix vs WT-mix	4	0.469	
	Seed capsules	population	Mix vs asLOX3	4	0.91	0
			WT vs asLOX3	4	**0.0001**	**2.583**
			WT vs Mix	4	**0.0002**	**1.150**
		genotype	asLOX3-mono vs asLOX3-mix	4	0.856	0.087
			asLOX3-mono vs WT-mix	4	0.688	0.068
			WT-mono vs asLOX3-mix	4	**0.001**	**1.419**
			WT-mono vs WT-mix	4	**0.005**	**0.937**
			asLOX3-mix vs WT-mix	4	0.6224	0.115
NI	Flowers	population	Mix vs asLOX3	4	0.706	
			WT vs asLOX3	4	0.239	
			WT vs Mix	4	0.415	
		genotype	asLOX3-mono vs asLOX3-mix	4	0.891	
			asLOX3-mono vs WT-mix	4	0.631	
			WT-mono vs asLOX3-mix	4	0.402	
			WT-mono vs WT-mix	4	0.626	
			asLOX3-mix vs WT-mix	4	0.762	
	Seed capsules	population	Mix vs asLOX3	4	**0.0004**	**0.910**
			WT vs asLOX3	4	**0.002**	**1.053**
			WT vs Mix	4	0.615	0.007
		genotype	asLOX3-mono vs asLOX3-mix	4	**0**	**0.889**
			asLOX3-mono vs WT-mix	4	**0.01**	**0.908**
			WT-mono vs asLOX3-mix	4	0.201	0.164
			WT-mono vs WT-mix	4	0.741	0.225
			asLOX3-mix vs WT-mix	4	0.166	0.330

There were differences between the two experiments regarding numbers of seed capsules. In the PM Experiment, when initial caterpillar density was not reduced and caterpillars were allowed to move among populations (Fig 1C), WT monocultures produced fewer seed capsules than other cultures, while mixtures and as*LOX3* monocultures did not differ significantly from each other (linear mixed-effects model, Table 4, Fig 5C). While WT plants in monocultures produced fewer seed capsules compared to both genotypes in mixture and as*LOX3* plants in monocultures, the number of seed capsules did not differ for as*LOX3* plants in monocultures from both genotypes in mixtures, or between genotypes within mixtures (linear mixed-effects model, Table 4, Fig 5D).

In the NI Experiment, when caterpillar density was reduced to mimic dispersal once larvae started to move among plants (Fig 1C), as*LOX3* monocultures produced fewer seed capsules than mixed and WT monocultures, while the number of seed capsules did not differ between mixed and WT monocultures (linear mixed-effects model, Table 3, Fig 5G). as*LOX3* plants in monocultures had fewer seed capsules than both genotypes in mixtures (linear mixed-effects model, Table 4, Fig 5H). The number of seed capsules did not differ between WT in monocultures and both genotypes in mixtures, or between genotypes within mixtures (linear mixed-effects model, Table 4, Fig 5H).

In summary, the number of flowers did not differ significantly by population or genotype in either experiment, and neither did plant size, but seed capsule number did. Mixtures showed stability in seed capsule production by having more consistent production compared to the two monocultures across experiments. Specifically, mixtures outcompeted WT monocultures under high caterpillar density (PM Experiment) and as*LOX3* monocultures under low caterpillar density (NI Experiment).

## Discussion

Plant genetic diversity can have cascading effects in ecological communities, but the mechanisms driving these effects remain largely unexplored. We manipulated a single key defense gene in experimental populations of *N*. *attenuata* which codes for a lipoxygenase enzyme providing substrate for the jasmonate (JA) plant defense hormones [31], and measured the resulting effects for herbivore communities and plant fitness correlates in a controlled, semi-natural setting (Fig 1). We chose to manipulate a key gene in JA biosynthesis because JA regulates many defense traits against herbivores, and variation in JA signaling and accumulation has been shown to alter herbivore community composition in wild *N*. *attenuata* populations [20,25]. Furthermore, JA signaling limits plant competitive growth and is an important regulator of potential trade-offs between plant growth and defense [41–43].

We exposed plant populations differing only in frequency of JA-deficient as*LOX3* genotypes (0%. 50% or 100%) to attack by three common native herbivores: *Empoasca* sp., *T*. *notatus* and *M*. *sexta* larvae, for which binary interactions are well characterized [20,21,26,27,32], providing the required mechanistic foundation for the community-level questions we ask in this study. We used two similar experimental designs, one which allowed observation of primarily plant-mediated effects (PM), and one which more accurately mimicked natural infestation (NI).

### WT plants in mixed culture were more heavily attacked by *Empoasca* sp.

*Empoasca* sp. is an opportunistic herbivore that mainly attacks plants in nature having lower JA production than their neighbors, and preferentially attacks as*LOX3* plants and other JA-deficient transgenic lines in experimental plantations [20,21]. Indeed, as*LOX3* monocultures were more damaged by *Empoasca* sp. than WT monocultures in the PM Experiment, and the same trend was apparent in the NI Experiment (Fig 3). Interestingly, mixtures comprising alternating WT and as*LOX3* plants in equal numbers were more damaged by *Empoasca* sp. than were WT monocultures, and damage rates did not differ between mixtures and as*LOX3* monocultures. This is because WT plants growing in the mixtures tended to be more damaged by *Empoasca* sp. than those growing in the monocultures. In addition, damage to WT plants growing in the mixtures was similar to that on neighboring as*LOX3* plants (Fig 3). The relatively high damage rate of *Empoasca* sp. on its uncommon host, WT, might have resulted from *Empoasca* sp. individuals moving between the two genotypes due to their close proximity in mixtures (Fig 1B), which reflects spatial distribution of *N*. *attenuata* plants clustered in native populations [6]. To detect JA-deficient plants, *Empoasca* sp. employs probing behavior with their stylets on the plants [20]. Therefore, when *Empoasca* sp. searches and probes new feeding leaves, the chance to feed on WT plants in the mixed culture due to the vicinity to neighboring as*LOX3* plants is high. Switching between as*LOX3* and WT in the mixtures probably results in the increased damage on the WT plants. It is likely that the presence of as*LOX3* plants in mixed population increases *Empoasca* sp. residence in this patch compared to WT monocultures. The effect size may depend on the density and frequency of as*LOX3* plants and neither factor was varied in this study. However, it is known that neighboring plants in mixed patches can influence interactions between focal plants and their herbivores, leading to associational resistance or associational susceptibility [44]. This depends on multiple factors, including the diversity of neighboring plants, herbivore movement and trait or plant density and frequency [45,46]. To investigate whether the higher damage rates of *Empoasca* sp. on WT plants in mixed populations are due to associational effects would require experiments that independently manipulate plant density, and frequency of JA-deficient plants.

Furthermore, in our study, we investigated the effect of manipulating a key plant trait on two herbivore traits, damage and growth. Consumer traits are a key component to understand the emergent pattern when manipulating plant diversity [47], especially traits related to feeding and movement behavior [9,48]. For example, mixing plant genotypes can reduce the efficiency of herbivore feeding. This was shown in a previous study, where the consumption by Japanese beetles (*Popillia japonica*) was reduced in genotypic mixtures of the common evening primrose (*Oenothera biennis*) compared to beetles feeding on plants of same genotype [49]. Although some studies showed that herbivore abundance might increase in response to increasing diversity, if the feeding efficiency is affected, the damage might not be positively correlated to an increase in herbivore abundance. Therefore, additional experiments focusing on herbivore traits in genetic mixtures vs monocultures are required to investigate *Empoasca* sp. feeding behaviors in these cultures. Monitoring movement and residence time on plant genotypes in small-scale experiments could reveal the pattern of host selection in mixed plant populations.

### *Empoasca* sp. infestation determined *T*. *notatus* damage

Interestingly, WT plants in mixtures received less damage from *T*. *notatus* than WT plants in monocultures, while as*LOX3* plants were attacked at a similar rate whether growing in monocultures or mixtures. *T*. *notatus* is a specialist herbivore that often colonizes *N*. *attenuata* plants in nature. *T*. *notatus* damage as well as many life history parameters are not affected by manipulating JA levels in host plants [27,30] although *N*. *attenuata* plants deficient in JA-regulated 17-hydroxygeranyllinalool diterpene glycosides (HGL-DTGs) receive more damage from *T*. *notatus* [50]. However, *N*. *attenuata* plants infested by *Empoasca* sp. receive less damage from *T*. *notatus* under natural conditions [30]. We found that under glasshouse conditions and in simplified experimental designs, plants co-infested with *Empoasca* sp. also received less damage by *T*. *notatus* (Fig 2A). Furthermore, both herbivores showed contrasting feeding preference within the plant: *Empoasca* sp. preferred older leaves, while *T*. *notatus* attacked younger leaves (Fig 2B). Finally, we can conclude that *Empoasca* sp. infestation rather than JA variation *per se* likely determined *T*. *notatus* damage patterns on *N*. *attenuata* plants [30].

A limited number of studies have addressed the effect of plant diversity on altering the interaction between herbivore species [9]. For example, plant-mediated indirect effects can alter herbivore interactions, such as colonization processes. While the specialist *T*. *notatus* can colonize both genotypes, in nature, *T*. *notatus* might simply disperse to colonize *Empoasca* sp.-free plants. Although colonization processes and herbivore movement are important factors, other subtle changes can also occur in response to variation in plant traits. For example, it was previously reported that co-infestation with *Empoasca* sp. shifts the female-biased sex ratio of *T*. *notatus* towards a higher proportion of male progeny, and this sex ratio shift can be attributed to changes in cytokinin-regulated plant traits such as nutrient quality that may be linked to *Empoasca* sp. co-infestation [27]. This could provide a mechanistic explanation as to why *T*. *notatus* avoids *Empoasca* sp.-damaged plants.

### *M*. *sexta* larval growth depended on larval density in mixtures

*M*. *sexta* larvae grew larger on as*LOX3* monocultures in comparison to WT monocultures in both experiments. This is consistent with previous literature that reported increased susceptibility of *LOX3-*silenced plants to attack by *M*. *sexta* larvae [31,51,52] and was independent of prior *T*. *notatus* damage. This is in contrast to work by Kessler and Baldwin [26] indicating that *T*. *notatus*-damaged *N*. *attenuata* plants are poor hosts for *M*. *sexta*, but part of the effect observed in that study was due to activity of native *Geocoris* spp. predators not present in the mesocosm experiments. Furthermore, in our study, all plants were damaged by *T*. *notatus*, and it is possible that the ca. 2-fold difference in percent canopy damage we observed did not result in differences in *M*. *sexta* growth. JA-deficient as*LOX3* plants accumulate less nicotine and trypsin protease inhibitors (TPI) as well as many other defensive metabolites in comparison to WT plants, and the reduced levels of these important direct defenses results in lower resistance to attack by *M*. *sexta* larvae [31,50,52].

However, mixtures showed different results under high or low caterpillar density. In the PM Experiment, caterpillar density was not reduced once larvae became mobile among plants of the same population, and caterpillar growth on mixtures was similar to that on as*LOX3* monocultures. This is likely explained by the movement of caterpillars among genotypes in mixtures. For example, caterpillars that first fed on as*LOX3* grew larger than those on WT plants and therefore, when they moved to a WT neighboring plant, likely removed more leaf area than the smaller caterpillars which had fed initially on WT. In the NI Experiment, we reduced caterpillar density once larvae became mobile among plants to mimic natural dispersal and predation, and growth on mixtures was found to be intermediate between that observed on WT and as*LOX3* monocultures. In addition, the damage caused by caterpillars to mixtures also tended to be intermediate. In both experiments, initial caterpillar growth depended on plant genotype identity rather than on whether the particular genotype was growing in mono- or mixed culture.

### Seed capsule production was more stable in mixtures

We counted flower number and seed capsule production as estimates of plant fitness [6,23]. Seed capsule production was lower for WT monocultures than as*LOX3* monocultures and mixtures when caterpillar density was high, and flower production showed the same tendency (PM Experiment). In the PM Experiment, caterpillars that fed on as*LOX3* monocultures and mixtures moved to WT monocultures once they had stripped the as*LOX3* plants of much of their foliage, and these WT monocultures were still coping with their own caterpillars. Under these conditions, WT plants in monocultures had to cope with the attack of multiple 4^th^ or 5^th^ instar caterpillars which removed a substantial portion of shoot biomass within a short time, leading to reduced seed production. WT plants in mixtures tended to produce more flowers and seed capsules than WT plants in monocultures. This may have been in response to increased competitive ability of as*LOX3* plants [33] and dilution of caterpillar attack, which was initially divided between the two genotypes. When caterpillar density was reduced to mimic dispersal out of the mesocosm (NI Experiment), the number of flowers did not differ by population type or genotype, but as*LOX3* monocultures produced fewer seed capsules than WT mono- and mixtures. In this experimental design, caterpillars were prevented from moving among populations by reducing their density and later removing them from the experiment. Therefore, WT plants in monocultures were exposed to fewer caterpillars in comparison to PM Experiment, mimicking a moderate attack scenario more commonly seen in nature. In addition, reducing caterpillar density and finally removing caterpillars from the experiment allowed WT plants to recover and produce more seed capsules in comparison to as*LOX3* monocultures, which tended to have more damage. The mixed culture probably had the advantage again of having caterpillars distributed among both genotypes, one of which is more susceptible but a better competitor.

JA induction plays an important role in mediating plant defense and associated fitness costs under herbivore attack [41,53–55]. The production of many JA-regulated specialized metabolites commands resources and signaling capacity that could be otherwise invested in reproduction, but has the fitness benefit of increasing plant resistance to herbivory [15,38,56–58] and consequently, the fitness of herbivores is negatively affected [55]. *M*. *sexta* attack results in immediate growth reduction of *N*. *attenuata* plants upon endogenous JA induction [59,60] as does treatment with methyl jasmonate (MJ) that results in slower growth of induced plants growing in competition with uninduced plants in the glasshouse [41] and in nature [42]. A combination of JA-regulated reconfiguration of general metabolism, induction of specialized metabolites that affect *M*. *sexta* larval growth [21,50,59,61,62], and different competitive ability among the genotypes account for the outcomes in both experiments: there were large differences in *M*. *sexta* damage but only small differences in patterns of *Empoasca* sp. and *T*. *notatus* damage between experiments. Mixtures outcompeted either WT or as*LOX3* under different herbivory regimes, as seed capsule production in WT and as*LOX3* monocultures showed stronger fluctuation across the two experiments. The fact that mixtures were not outcompeted by both monocultures under different herbivory regimes can be interpreted as form of stability. When herbivore intensity varies throughout and between seasons, having a consistent performance compared to other populations may ensure sufficient representation of seeds over multiple generations in seedbanks and in the next growing season. Thus the Darwinian fitness of different genotypes may depend on their frequency in populations or patches. In summary, plant competitive ability versus resistance to attack, as well as diluted attack due to the mixing of two genotypes, could account for the seed capsule production in the mixed culture under different herbivory regimes.

The diversity-stability hypothesis predicts that biodiversity increases stability of ecosystem processes against perturbations, but in some cases opposite effects were detected [63,64]. A large body of literature has theoretically or experimentally investigated effects of diversity on different kinds of stability, most prominently temporal stability, resistance and resilience (recovery) due to diversity as measured by species richness, functional traits, functional groups or genotypes in communities [65–70]. Previous studies have helped to refocus the biodiversity-ecosystem function debate and highlighted the consequences for ecosystem function triggered by altering levels of biodiversity, especially under perturbation regimes or changing environments. However, the results of manipulating biodiversity were often explained by abstract concepts rather than measurable mechanisms, and these concepts were not always experimentally demonstrated. For example, at least some species or individuals with particular functional traits will show differences in response to perturbations in diverse communities [71]. It has been proposed that stability might result from reducing competition [64] or from facilitation in diverse communities [72]. However most of these studies have not measured competition or facilitation. When species-level or uncharacterized genetic biodiversity is manipulated, many unknown factors or traits that are associated with the studied trait may contribute to observed effects. In contrast, manipulating functional diversity by altering a single key gene increases mechanistic resolution, providing more predictive power. In our study, stability in plant reproductive output measured as more consistent performance in mixtures resulted from the differences of genotype performance when growing in mono- or mixtures under different levels of herbivore attack. This is correlated to both the plant’s competitive ability and susceptibility to herbivores, which are regulated by varying JA production via *LOX3* expression.

Variation in plant defense traits can strongly affect interactions and performance of herbivore populations. Also, variability in plant nutrient levels is essential part of this interaction. A previous meta-analysis showed that variability in nutrient levels could suppress herbivore populations [73]. However, it is challenging to separate the contribution of plant nutritive versus defensive traits. Key gene might be a valuable tool to separately manipulate nutritive and defensive traits to test their relevance.

### Summary and outlook

Our data shows that variation in plant defense traits can directly affect host selection and herbivore performance in mixed populations. We also show that other indirect consequences of varying plant genotype, for example increased damage from one herbivore species in the community, can alter damage rates of another herbivore species. Furthermore, we found seed capsule output in mixtures was more stable under high or low density of *M*. *sexta* caterpillars. Susceptibility to herbivores and plant competitive ability are oppositely mediated by JA-regulated traits. These data indicate the usefulness of a key gene approach to link variation in plant defense traits to herbivore interactions and the consequences for plant populations in community- level study. Future studies could profit from such approach to test specific predictions about the consequences of varying specific traits.

Moving the field of ecological research towards a more predictive science is essential to cope with environmental change scenarios [74]. Investigating functional traits controlled by known key genes is an approach that might help in predicting interaction patterns in ecological communities, and generating communities with predictable responses to perturbation. This perspective is also relevant in applied ecology and agricultural systems. Genetically diverse crop systems rely on traditional varieties often used in small scale farms [75] which deliver multiple benefits such as increased yield stability, productivity, reduced pest load and improved soil fertility [76]. In contrast, industrial agriculture relies heavily on monocultures, which, for example, minimize differences in harvesting time that result from combining multiple cultivars. A key genes approach could provide a tool for large scale agriculture to develop functionally more diverse crops while avoiding unwanted variation, hopefully limiting the tradeoff between preserving biodiversity and satisfying rapidly increasing food demands.

This approach does require that organisms are genetically characterized or closely related to characterized organisms. Also, it relies on previous knowledge of gene function or biosynthetic pathways and therefore, uncharacterized key genes are of little value for this approach [77]. However, natural variation in phenotypes with heritable genetic bases that support communities might be targeted and tested in controlled experimental designs. Genome editing and cheap genome sequencing will facilitate gene manipulation in a growing number of organisms. Finally, future research might focus on the role of key genes in natural environments [55] to reveal insights into emergent properties resulting from the interaction of diverse plant populations with multiple biotic and abiotic stresses.

## Supporting information

S1 File Damage of *Empoasca* sp. and *Tupiocoris notatus*; data to show that *Manduca sexta* caterpillar growth was reduced on WT in comparison to as*LOX3* plants; soil layers, soil components, and fertilization parameters in the mesocosm; and measures of different plant growth parameters. (PDF)

S2 File Soil moisture and temperature measurement in the mesocosm. (ZIP)
