# Supplementary material for: Functional variation in a key defense gene structures herbivore communities and alters plant performance
Source: PLoS One. 2018 Jun 6;13(6):e0197221. doi: 10.1371/journal.pone.0197221 (PMC5991399; doi:10.1371/journal.pone.0197221)
Supplement: S1 File — (PDF) [file pone.0197221.s001.pdf]

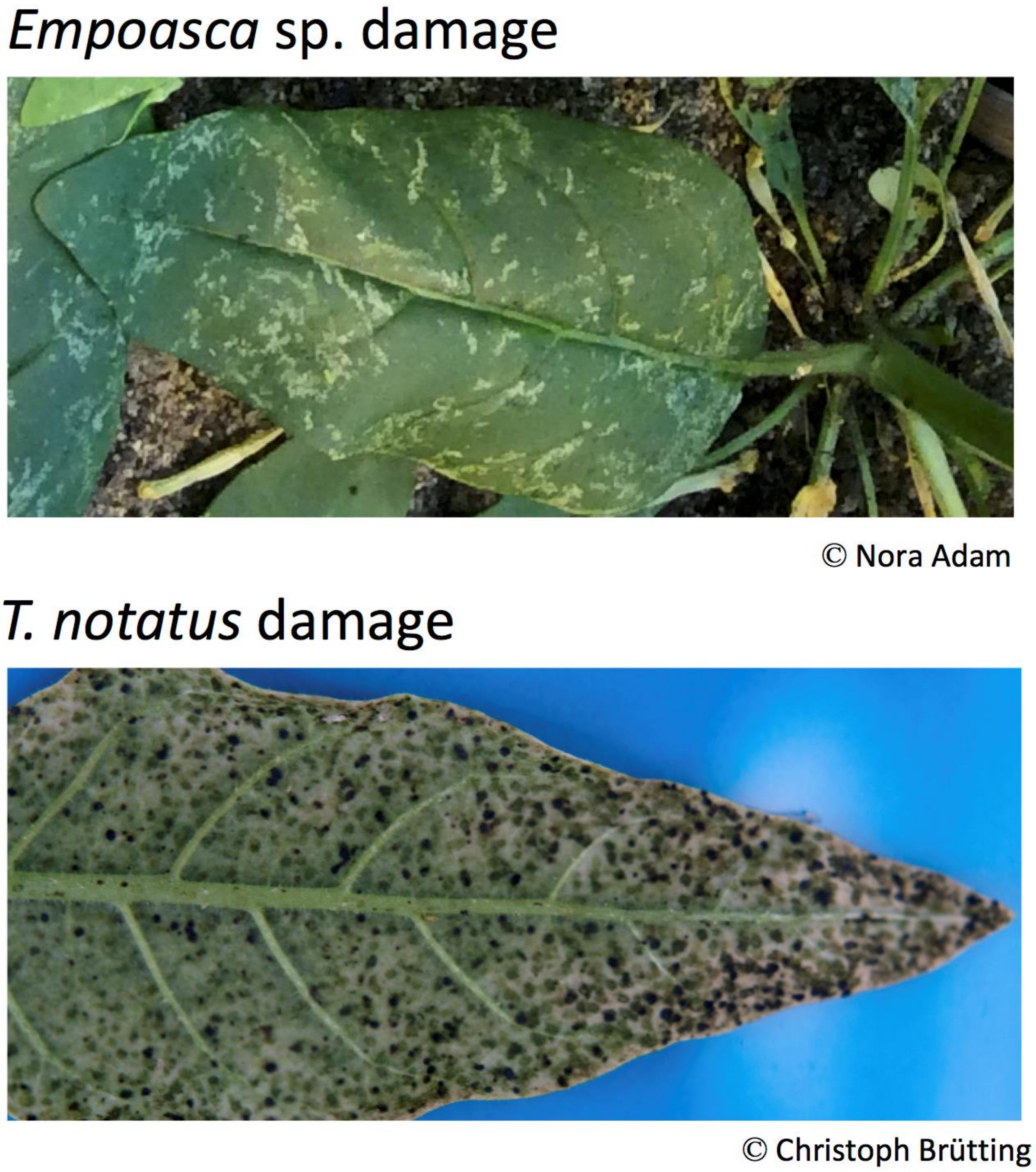

Figure A. Damage of *Empoasca* sp. and *Tupiocoris notatus*.

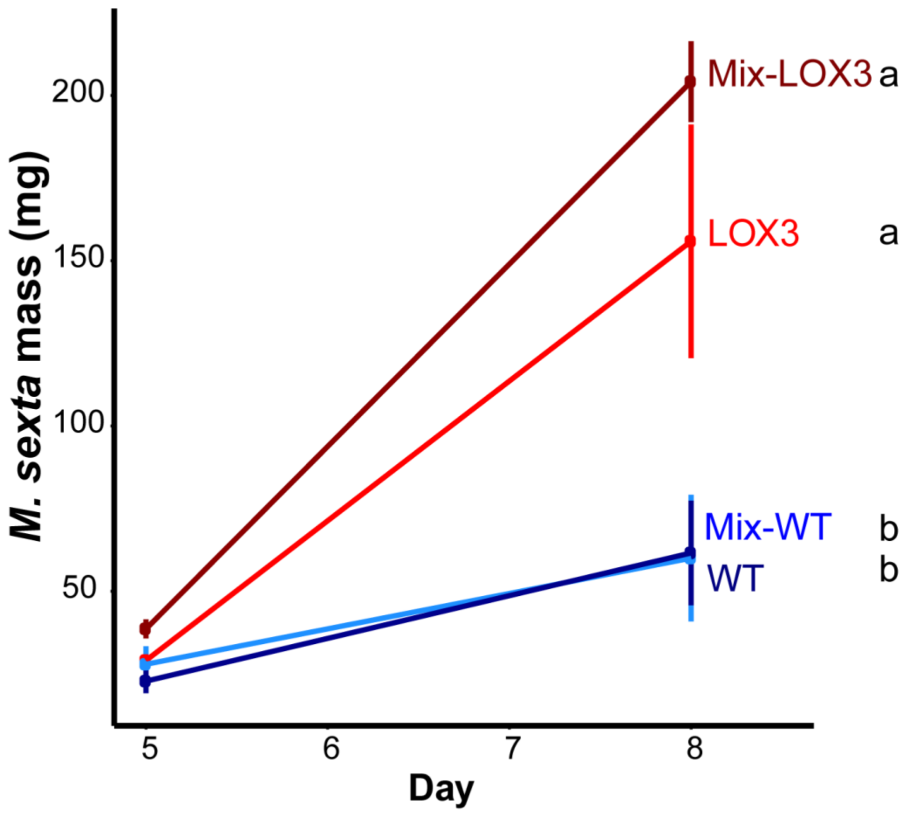

Figure B. *Manduca sexta* caterpillar growth was reduced on WT in comparison to as*LOX3* plants.

Table A. Soil layers in the mesocosm

	L/container
**A - Upper layer** (30cm)	
Nicotiana - Substrate	48
Sand aggregate 0.7-1.2 mm	24
Sand aggregate 0.4-0.8 mm	24
Klasmann-clay granules	24
**B - Middle layer** (30cm)	
Nicotiana – Substrate	40
Sand aggregate 0.7-1.2 mm	80
**C - lower layer** (30cm)	
Nicotiana – Substrate	40
Sand aggregate 0.7-1.2 mm	40
Lavabims 8-16mm sand aggregate	40
**Drainage layer** (4th and 5th layers)	
**D** - Lecaton- Hydrogranulate 2-4 mm (2cm)	8
**E** - Lecaton- Hydrogranulate 8-16 mm (10cm)	38
**Total**	**406**

Table B. Soil components in the mesocosm

Material	Contents			Nutrients[mg/l] N	P_2O_5	K_2O	PH	Salt content [g/l]	Manufacturers	Address
**Fruhstorfer**									Fruhstorfer Erde	Langer Damm 1, 49377 Vechta
**Nullerde**	raised-bog peat, Vulkanton			20-40	20-40	40-60	5.5-6.0	<0.5	HAWITA GRUPPE GmbH	
					10-17 (P)	*33-50 (K)*				
Quartz sand		H_2-H_4^*	H_4-H_6^*							
Grain diameter [mm]		0.4-0.8	0.7-1.2							
**Clay granules Lamstedt**										
Clay granules 2-4 mm							9.6 (CaCl)	0.9 (KCl)	Fibo Ex Ciay Deutschland GmbH	Rahdener Strasse1, 21769 Lamstedt,
Clay granules 8-16 mm									Gärtnereibedarf Kammlott GmbH	Bergrat-Voigt-Straße 1, 99087 Erfurt
**Klasmann substrate**									Klasmann - Deilmann GmbH	Postfach 1250, 49741 Geeste-Gross Hesepe
Clay granules 2 - 8 mm	65% clay mineral							7.7		
	(Smectit/Vermiculite, Illite, Kaolinite) 35% other minerals									
	(Quart, Feldspar, Mica)									
	SiO_2		66%							
	Al_2O_3		15.10%							
	Fe_2O_3		6.10%							
	K_2O		3.60%							
	MgO		1.60%							
	CaO		1.20%							
8-16 mm expanded slate sand aggregate									ulopor Thüringer Schiefer GmbH	OT Unterloquitz Neustedt 21, 07330 Probstzella
Drainage layer expanded slate									Optigrün international GmbH	Am Birkenstock 19, 72505 Krauchenwies-Göggingen

* Decomposition rate

Table C. Fertilization parameters in the mesocosm.

Fertilizer	Contents (%)												Manufacturers	Address
	N	P_2O_5 (P 43,6%)*	K_2O (K83,3%)*	MgO	S	B	Cu	Fe	Mn	Mo	Zn	CaO		
**Epsotop** (Magnesium sulfate)				16	13								K+S Kali GmbH	Bertha-von-Suttner-Str. 7, 34131 Kassel
**Micromax** (0.3-0.5 g /L substrate)					15	0.1	0.5	12	2.5	0.05	1		Scotts Deutschland GmbH	48527 Nordhorn
**Superphosphate**		20 (8.7)											Hi-Chem nv	Generaal de Wittelaan 17 bus 16, B 2800 Mechelen/ Belgien
**Multi Mix substrate fertilizer**	14	16	18	0	0	0.03	0.15	0.09	0.16	0.05	0.04		Haifa Chemicals Ltd.	P.O. Box 10809, Haifa Bay 26120 ISRAEL
Nitrate	5.2													

* Conversion factor

Table D. Measures of different plant growth parameters.

		Population level			Genotype level	
**Parameter**	**Experiment**	as*LOX3*	Mix	WT	as*LOX3*–Mix	WT–Mix
Rosette diameter	PM	31.7 ± 0.96	31.46 ± 0.73	26 ± 2.86	31.43 ± 0.38	31.5 ± 1.25
(cm)	NI	28.4 ± 0.85	29.65 ± 2.38	30.91 ± 2.80	29.90 ± 2.70	29.40 ± 2.07
Height (cm)	PM	53.22 ± 2.92	47.9 ± 5.63	42.99 ± 4.81	50.77 ± 4.46	45.04 ± 6.44
	NI	82.84 ± 3.61	86.65 ± 9.14	86.91 ± 9.57	87.89 ± 10.13	85.41 ± 8.35
Fresh weight (g)	PM	22.73 ± 2.50	23.48 ± 2.40	24.22 ± 11.6	22.17 ± 5.06	24.79 ± 1.59
	NI	18.33 ± 1.83	21.05 ± 1.55	21.31 ± 3.23	20.68 ± 1.77	21.42 ± 1.36
Dry weight (g)	PM	3.59 ± 0.54	3.92 ± 0.31	3.16 ± 0.8	4.23 ± 0.52	3.61 ± 0.23
	NI	2.85 ± 1.25	5.19 ± 1.32	5.77 ± 1.98	5.19 ± 1.57	5.19 ± 1.22

Rosette diameter, plant height and biomass in Plant-Mediated (PM) and Natural Infestation (NI) Experiments (mean ± SEM, n = 4 populations, each of which represents the mean of 4-8 plants)
