# Supplementary material for: Functional variation in a key defense gene structures herbivore communities and alters plant performance
Source: PLoS One. 2018 Jun 6;13(6):e0197221. doi: 10.1371/journal.pone.0197221 (PMC5991399; doi:10.1371/journal.pone.0197221)
Supplement: S2 File — (ZIP) [file pone.0197221.s002.zip › S2_File/Mesocosm/Mesocosm1.html]

"Mesocosm1"


Graph selection
cylinder1: 
  
cylinder2: 
  
cylinder3: 
  
cylinder4: 
  
cylinder5: 
  
cylinder6: 
  
temperature: 
"Mesocosm1"
  
  
temperature sensor in cylinder 3
  
  
  
  
