# Supplementary material for: Functional variation in a key defense gene structures herbivore communities and alters plant performance
Source: PLoS One. 2018 Jun 6;13(6):e0197221. doi: 10.1371/journal.pone.0197221 (PMC5991399; doi:10.1371/journal.pone.0197221)
Supplement: S2 File — (ZIP) [file pone.0197221.s002.zip › S2_File/Mesocosm/Mesocosm3.html]

"Mesocosm3"


Graph selection
temperature: 
  
humidity: 
  
light: 
  
"Mesocosm3"
  
  
  
  
