# Supplementary material for: Functional variation in a key defense gene structures herbivore communities and alters plant performance
Source: PLoS One. 2018 Jun 6;13(6):e0197221. doi: 10.1371/journal.pone.0197221 (PMC5991399; doi:10.1371/journal.pone.0197221)
Supplement: S2 File — (ZIP) [file pone.0197221.s002.zip › S2_File/Sensoren Mesocosm.docx]

Sensoren Mesocosm

Bodenfeuchtigkeit:

- Bodenfeuchtigkeit (Vegetronix VH400) von DVS Beregnung
- Gibt ein Signal aus von 0-3V (0V Trocken ; 3V Wasser)
- Bodenfeuchtigkeit in 10cm tiefe

Bodentemperatur

- DS18B20 digitaler Temperatursensor in 10cm tiefe
- Von EXP-tech (EXP GmbH)

Temperatur & Luftfeuchtigkeitssensor

- Sensirion SHT75 (digitaler T&F Sensor)

Lichtsensor

# Digitaler Lichtsensor (TSL2591)

- Gibt die Lichtmenge in Lux aus
